# The serine protease inhibitor serpinE2 is a novel target of ERK signaling involved in human colorectal tumorigenesis

**DOI:** 10.1186/1476-4598-9-271

**Published:** 2010-10-13

**Authors:** Sébastien Bergeron, Etienne Lemieux, Véronique Durand, Sébastien Cagnol, Julie C Carrier, Jacques G Lussier, Marie-Josée Boucher, Nathalie Rivard

**Affiliations:** 1 Department of Anatomy and Cellular Biology, CIHR Team on Digestive Epithelium, Faculty of Medicine and Health Sciences, Université de Sherbrooke, Sherbrooke, Québec, Canada; 2 Service of Gastroenterology, Department of Medicine, Faculty of Medicine and Health Sciences, Université de Sherbrooke, Sherbrooke, Québec, Canada; 3 Faculté de Médecine Vétérinaire, Université de Montréal, St-Hyacinthe, Québec, Canada

## Abstract

**Background:**

Among the most harmful of all genetic abnormalities that appear in colorectal cancer (CRC) development are mutations of KRAS and its downstream effector BRAF as they result in abnormal extracellular signal-related kinase (ERK) signaling. In a previous report, we had shown that expression of a constitutive active mutant of MEK1 (caMEK) in normal rat intestinal epithelial cells (IECs) induced morphological transformation associated with epithelial to mesenchymal transition, growth in soft agar, invasion and metastases in nude mice. Results from microarrays comparing control to caMEK-expressing IECs identified the gene encoding for serpinE2, a serine protease inhibitor, as a potential target of activated MEK1.

**Results:**

1- RT-PCR and western blot analyses confirmed the strong up-regulation of serpinE2 expression and secretion by IECs expressing oncogenic MEK, Ras or BRAF. 2- Interestingly, serpinE2 mRNA and protein were also markedly enhanced in human CRC cells exhibiting mutation in *KRAS *and *BRAF*. 3- RNAi directed against serpinE2 in caMEK-transformed rat IECs or in human CRC cell lines HCT116 and LoVo markedly decreased foci formation, anchorage-independent growth in soft agarose, cell migration and tumor formation in nude mice. 4- Treatment of CRC cell lines with U0126 markedly reduced *serpinE2 *mRNA levels, indicating that expression of *serpinE2 *is likely dependent of ERK activity. 5- Finally, Q-PCR analyses demonstrated that mRNA levels of serpinE2 were markedly increased in human adenomas in comparison to healthy adjacent tissues and in colorectal tumors, regardless of tumor stage and grade.

**Conclusions:**

Our data indicate that serpinE2 is up-regulated by oncogenic activation of Ras, BRAF and MEK1 and contributes to pro-neoplastic actions of ERK signaling in intestinal epithelial cells. Hence, serpinE2 may be a potential therapeutic target for colorectal cancer treatment.

## Background

Colorectal cancer (CRC) is the second leading cause of cancer-related deaths in North America. Number of genetic and epigenetic alterations has been reported to be involved in colorectal tumorigenesis, such as chromosome instability, DNA methylation, gene amplification and mutation. *APC *is the most frequently mutated gene (53.8%), followed by TP53 (37.2%) and the two members of the MAPK pathway, KRAS (35.1%) and BRAF (10%) [1]. In this regard, aberrant activation of the Ras/Raf/MEK/ERK pathway leads to the downstream activation of MEK1/2 and ERK1/2 kinases, which may control many features of tumorigenesis [2]. In keeping with this observation, we and others have recently shown that expression of constitutively active MEK1 in non-transformed rodent intestinal epithelial crypt cell lines is sufficient to induce growth factor relaxation for DNA synthesis and to promote morphological transformation and growth in soft agar [3,4]. Accordingly, it has been demonstrated that MEK is phosphorylated and activated in 30-40% of adenomas and in 76% of colorectal tumors [5,6]. CRCs also exhibit particularly high frequencies of ERK activation [7] and some studies have reported that ERK1/2 activities are indeed elevated in intestinal tumors [8,9]. Therefore, much emphasis has been placed on treatment strategies that target this protein kinase cascade [10]. In particular, potent and selective inhibitors of MEK1 and MEK2 have been developed and have been tested in phase I/II clinical trials (AZD6244, XL51, and ARRY-162) [11,12]. Interestingly, an early study reported that the enzymatic activity of ERK1/ERK2 is markedly up-regulated during late progression of carcinogen-induced colon carcinomas [13]. In this respect, activation of MEK1 and MEK2 in intestinal epithelial cells is sufficient to induce invasive and metastatic tumors in nude mice [14,15]. Together, these observations strengthen the notion that ERK1/2 MAP kinase signaling may play a critical role in CRC progression [16]. However, in spite of the obvious role of MEK/ERK kinases in the induction and regulation of intestinal epithelial cell transformation, tumorigenesis and metastasis, little is known regarding the molecular mechanism by which MEK/ERK signaling achieves such functions.

In order to further understand the mechanisms by which activated MEK1 induces tumorigenesis in intestinal epithelial cells, we have analyzed by microarray the pattern of gene expression in intestinal epithelial (IEC-6) cells overexpressing activated MEK1. Importantly, *Serpin clade E member 2 *(*SerpinE2*), emerges as the highest up-regulated gene induced by activated MEK1. Serpins are SERine Protease INhibitors targeting proteases prostatin [17-19], matriptase [20], T cell proteinase-1 [21], trypsin, thrombin, plasmin and plasminogen activator [22,23]. Through their ability to reduce proteolysis, serpins are predicted to impair extracellular matrix degradation and consequently cancer cell invasion and metastasis. However, serpinE1 (or plasminogen activator inhibitor-1, PAI-1) has been reported to promote angiogenesis and to induce tumor cell migration [24-26] while serpinE2 (or protease nexin-1, PN-1) appears to enhance the invasive potential of pancreatic [27], breast [28] and lung cancer cells [29]. Furthermore, serpinE1 is overexpressed in highly aggressive human breast tumors while serpinE2 levels are elevated in pancreatic tumors [27], breast tumors [28], oral squamous carcinomas [30], liposarcomas [31] and more recently CRCs [32].

In the present study, we show that RNA interference (RNAi) targeting *serpinE2 *in MEK1-transformed rat IECs or in human colorectal cancer cells decreased anchorage independent growth, migration and tumor formation in nude mice. Furthermore, serpinE2 is overexpressed in human adenomas and colorectal tumors compared to the adjacent healthy tissues. Therefore, our results demonstrate an important role for serpinE2 in colorectal tumorigenesis.

## Results

### SerpinE2 is overexpressed in intestinal epithelial cells transformed by activated MEK1 and oncogenic RAS and BRAF

**Figure 1:**
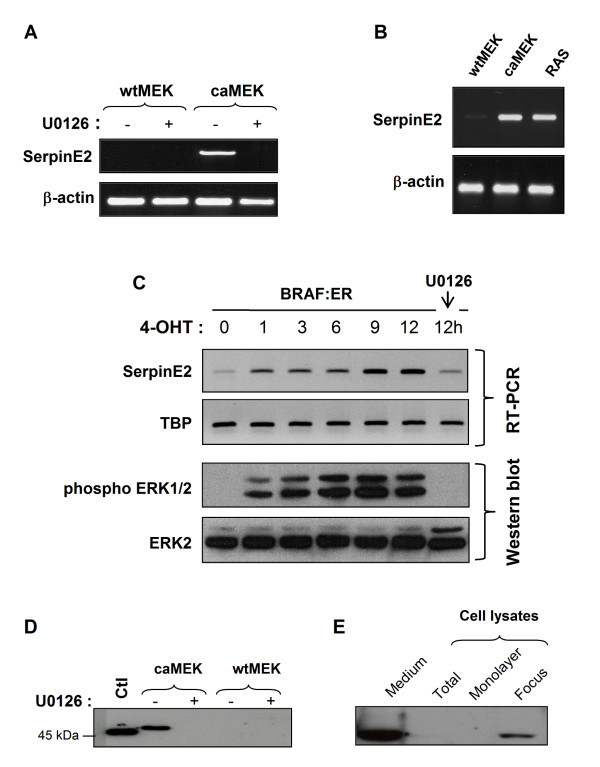
**SerpinE2 is overexpressed in intestinal epithelial cells transformed by oncogenic MEK1, RAS and BRAF**. **A- **wtMEK- and caMEK-expressing IECs were treated or not with U0126 (20 μM) during 24 h. Thereafter, cells were lysed and RNA isolated for *serpinE2 *or *β-actin *gene expression by RT-PCR. **B- **IEC-6 expressing oncogenic RAS or wtMEK or caMEK were lysed and RNA isolated for *serpinE2 *or *β-actin *gene expression by RT-PCR. **C- **BRAF:ER cells were stimulated with 250 nM 4-hydroxy-tamoxifen (4-OTH) for the indicated time periods. Thereafter, cells were lysed and RNA isolated for *serpinE2 *or *TATA-binding protein *(TBP) gene expression by RT-PCR. For the 12 h time point, cells were also treated with 20 μM U0126. ERK1/2 phosphorylation levels and total ERK1/2 were monitored by Western blot. **D- **wtMEK- and caMEK-expressing cells were treated or not with U0126 (20 μM) during 24 h. Thereafter, equal amounts of concentrated culture medium from wtMEK- and caMEK-expressing cells were analyzed by Western blotting with specific antibodies against serpinE2 as described in Material and Methods. Bovine follicular fluid was used as a positive control (CTL). **E- **caMEK-expressing IECs were cultured at post-confluence, lysed in Laemmli buffer and analyzed by Western blotting with specific antibodies against serpinE2 (lane 2). The culture medium was concentrated and also analyzed by Western blotting for serpinE2 expression (lane 1). In some experiments, foci were harvested by aspiration with a pipet, lysed in Laemmli buffer (lane 4) and analyzed by Western blotting for the expression of serpinE2. The surrounding cells (monolayer, lane 3) were also lysed in Laemmli buffer for Western blotting with specific antibodies against serpinE2.

Among the most harmful of all genetic abnormalities that appear in CRC development are mutations of KRAS and its downstream effector BRAF as they result in abnormal ERK signaling. In a previous report, we had shown that expression of a constitutive active mutant of MEK1 (caMEK) in the intestinal epithelial cell line IEC-6 induced morphological transformation and growth in soft agar; in marked contrast, wtMEK overexpression had no effect on IEC-6 phenotype [3]. In order to understand the mechanisms by which activated MEK1 induces intestinal cell tumorigenesis, the pattern of gene expression was analyzed by microarray in IEC-6 cells overexpressing activated MEK1. Results from microarrays comparing control (wtMEK) to caMEK-expressing IEC-6 cells identified the *Serpin clade E member 2 *(*serpinE2 *or *PN-1*) gene as a potential target of activated MEK1. Indeed, *serpinE2 *expression was significantly induced by more that 28-fold (p < 0.05) in cells overexpressing activated MEK1 in comparison to cells expressing wtMEK (data not shown). Overexpression of *serpinE2 *in caMEK-expressing IECs was furthermore confirmed following RT-PCR analysis as shown in Figure 1A. SerpinE2 expression was also markedly enhanced in IEC-6 cells transformed by oncogenic RAS (26-fold) or BRAF (12-fold after 12 h of 4-hydroxytamoxifen (4-OHT) (Figure 1B and 1C). Of note, the induction of serpinE2 was induced within 1 h following ERK activation as observed in cells expressing the inducible BRAF:ER fusion protein stimulated with 4-OHT (Figure 1C). Treatment with the MEK-inhibitor U0126 completely abrogated serpinE2 gene expression induced by oncogenic MEK1 (Figure 1A) and BRAF (Figure 1C), indicating that induction of serpinE2 is an early and direct event occurring following the activation of ERK signaling.

Since serpinE2 protein is known to be secreted [22,33], we easily confirmed its presence in conditioned culture medium of caMEK-expressing IECs whereas no serpinE2 protein was detected in the culture medium of wtMEK-expressing or parental IECs (Figure 1D). Again, treatment with the MEK-inhibitor U0126 completely abrogated serpinE2 secretion (Figure 1D). Interestingly, serpinE2 protein was difficult to detect in total cell lysates (Figure 1E, lane 2). However, serpinE2 was easily observed in lysates prepared from foci of post-confluent caMEK-expressing cells (Figure 1E, lane 4), while it was not detectable in the surrounding monolayer (Figure 1E, lane 3). This indicates a stronger expression of serpinE2 protein by the transformed IECs forming the foci.

### Gene silencing of serpinE2 decreases foci formation, growth in soft agarose and migration induced by activated MEK

**Figure 2:**
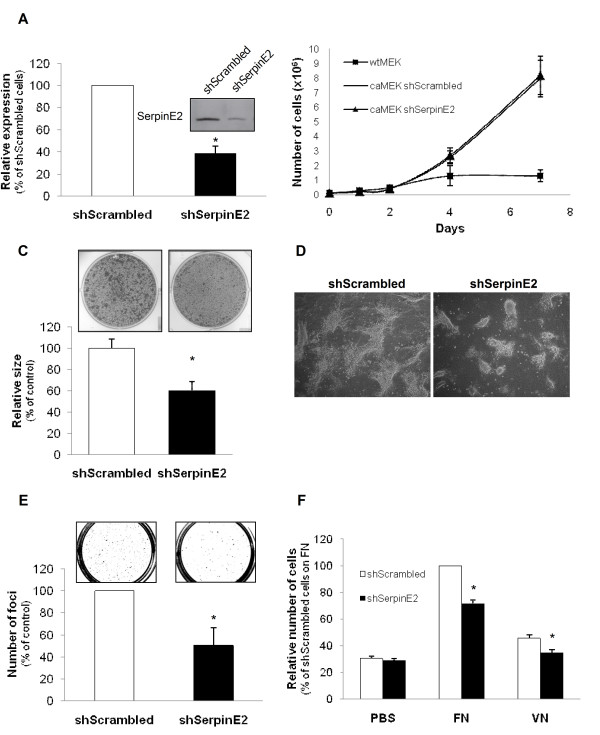
**SerpinE2 silencing in caMEK-expressing IECs decreases foci formation, growth in soft agar and migration**. **A- **caMEK-expressing IECs were stably infected with lentiviruses encoding for a control shRNA (scrambled sequence) or encoding a serpinE2-specific shRNA. These stable cell populations were thereafter lysed at confluence and equal amounts of concentrated culture medium were analyzed by Western blotting with specific antibodies against serpinE2. The graph illustrates densitometric analysis performed with the western blot data shown on the left to determine the % of serpinE2 downregulation. The level of serpinE2 observed in shScrambled cells was set at 100%. A representative experiment of three independent experiments is shown. **B- **wtMEK/IEC-6 or caMEK/IEC-6 cells expressing either shScrambled or shSerpinE2 were seeded in a 6-well plate at 100 000 cells per well. Cells were harvested and counted. Values are means of 4 experiments ± SE. **C and D- **caMEK-transformed cells expressing or not shSerpinE2 were seeded on parental IEC-6 cell monolayer during 15 days. Thereafter, the cells were stained with crystal violet and images of colonies were acquired under light microscopy. The size of the foci was calculated using Image J software and expressed as % of shScrambled cells (control) which was set at 100%. **E- **Cells were cultured in soft agarose for 3 weeks before MTT. The number of colonies was determined using Image J software and expressed as % of shSrcambled cells (control) set at 100%. **F- **Cell migration to the undersurface of the polycarbonate membrane of Boyden chambers coated with fibronectin (FN) or vitronectin (VN) was evaluated 24 h after seeding, in presence of 20 mM hydroxyurea. Values were expressed as a % of shScrambled cells migrating on fibronectin. *, significantly different from shScrambled cells at p < 0.05 (Student's *t *test).

In order to determine the contribution of serpinE2 in intestinal transformation induced by activated MEK, foci from post-confluent caMEK-expressing IECs were retrieved by aspiration with a pipette and pooled as one caMEK-expressing cell population. All further experiments were performed with this previously characterized caMEK-expressing IEC population [14] and compared with wtMEK-expressing cell populations. Recombinant lentiviruses encoding anti-*serpinE2 *short hairpin RNA (shRNA) were therefore developed to stably suppress serpinE2 levels in these cells. Several lentiviral constructs were generated and tested for their ability to knock down serpinE2 protein. One of these viral shRNAs was selected and designated as shSerpinE2. caMEK-expressing cells were henceforth infected with shSerpinE2 lentiviruses or with lentiviruses expressing a control shRNA (shScrambled). Secretion of serpinE2 protein was analyzed 14 days after selection with blasticidin S in these populations. As shown in Figure 2A, secreted serpinE2 levels were markedly reduced (> 60%) in cells-expressing shSerpinE2; in contrast, shScrambled had no effect on the secretion of serpinE2 (data not shown).

To determine the functional role of serpinE2 in caMEK-expressing cells, the proliferation rate of these cell populations was assessed when cultured on plastic. No difference was observed in the proliferation rate of subconfluent caMEK-expressing cells when serpinE2 expression was downregulated (Figure 2B). In a previous study, we had shown that expression of activated MEK in intestinal epithelial cells resulted in loss of cell-cell contact growth inhibition and produced colonies or multilayered domes which grew to increased saturation density and formed tumors when transplanted into nude mice [14]. Of note, focus formation assays performed herein revealed that initially, there was little difference in the number of foci obtained between control cells and serpinE2-depleted cells (data not shown). However, *serpinE2 *silencing markedly reduced the size of foci (Figure 2C) suggesting a reduced capacity of these foci to grow. Indeed, phase-contrast microscopy revealed that the colonies were smaller when serpinE2 was downregulated (Figure 2D). Finally, expression of shSerpinE2 led to a significant decrease in the ability of caMEK-expressing cells to grow under anchorage-independent conditions in soft agarose (Figure 2E).

Cell migration is an important process of tumorigenesis and metastasis. Moreover, we recently reported that intestinal epithelial cells expressing activated MEK1 clearly acquire an increased capacity to migrate as compared to wtMEK-expressing cells [14]. Herein, in an *in vitro *transwell migration assay, serpinE2 deficiency significantly reduced caMEK-expressing IEC migration to the undersurface of the polycarbonate membrane of Boyden chambers coated with fibronectin or vitronectin (Figure 2F), two extracellular matrix proteins which can interact with serpinE2 [34,35]. Taken together, these results support a role of serpinE2 in MEK1-induced transformation whereby serpinE2 activates anchorage-independent growth and cell migration.

### Expression of serpinE2 in colorectal cancer cells is dependent on MEK/ERK activity

**Figure 3:**
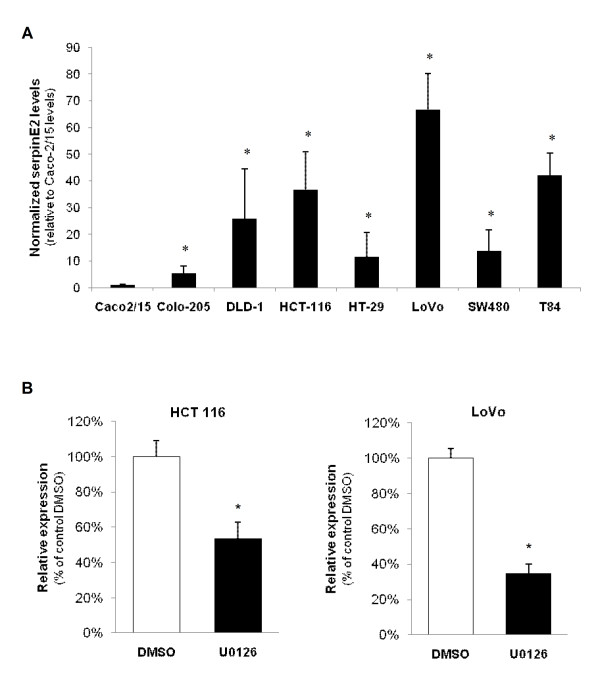
**Expression of serpinE2 in colorectal cancer cells is dependent on MEK/ERK activation**. **A- **Total RNA was isolated from CRC cell lines and processed for Q-PCR to analyze *serpinE2 *gene expression as described in Material and Methods. The relative level of each RNA was calculated using the standard curve method and normalized to the corresponding β2MIC RNA level. *, significantly different from Caco-2/15 (set at 1) at p < 0.01 (Student's *t *test). **B- **HCT116 and LoVo cells were treated daily with U0126. After two days, cells were lysed and total RNA was isolated for Q-PCR for *serpinE2 *gene expression. The relative level of each RNA was calculated using the standard curve method and normalized to the corresponding β2MIC RNA level. *, significantly different from control (DMSO) at p < 0.05 (Student's *t *test).

To assess the contribution of serpinE2 in human colorectal cancer, serpinE2 expression was first examined in various CRC cell lines including Caco-2/15 as well as others exhibiting mutation in *KRAS *(HCT-116, DLD-1, LoVo, SW480, T84) or *BRAF *(Colo-205, HT-29) [36]. As shown in Figure 3A, *serpinE2 *mRNA levels were barely detectable in the Caco-2/15 cell line while being markedly expressed in all other CRC cell lines tested. Two human CRC cell lines, namely HCT116 and LoVo, which have an activating mutation in the *KRAS *gene resulting in elevated MEK/ERK activities [37], were thereby chosen to further analyze the regulation and role of serpinE2 expression in human colorectal cancer cells. In addition, the impact of U0126 treatment was also investigated to evaluate the contribution of endogenous MEK/ERK activities in serpinE2 expression in human cell models. Forty-eight-hour treatment of HCT116 and LoVo cell lines with U0126 efficiently blocked endogenous MEK activity as confirmed by the marked inhibition of ERK1/2 phosphorylation (data not shown) [14]. As shown in Figure 3B, treatment of these CRC cell lines with U0126 markedly and significantly reduced *serpinE2 *mRNA levels, indicating that expression of *serpinE2 *is likely dependent of ERK activity in these cell lines.

### Down-regulation of serpinE2 expression in human colorectal cancer cells inhibits soft agarose colony formation, migration and tumor growth in nude mice

**Figure 4:**
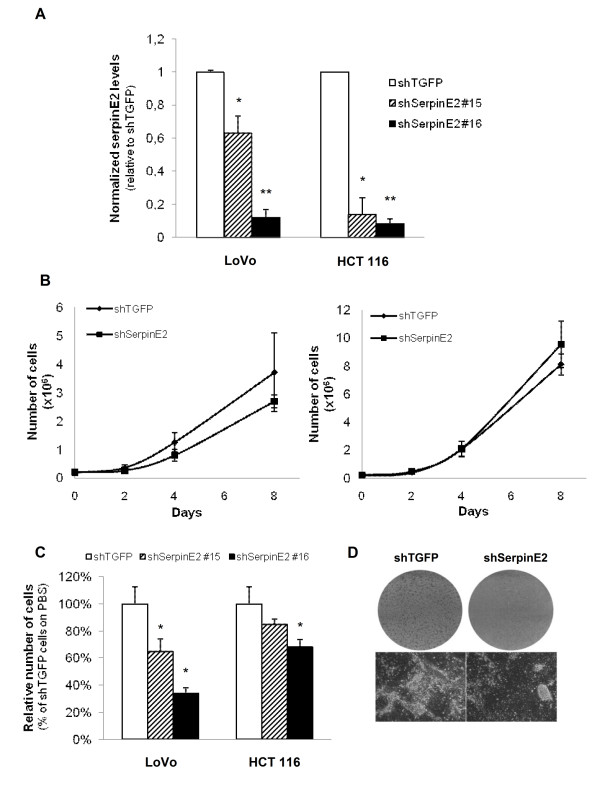
**Down-regulation of serpinE2 expression in human colorectal cancer cells inhibits colony formation**. **A- **HCT116 and LoVo cells were stably infected with lentiviruses encoding for a control shRNA (against TGFP) or encoding serpinE2-specific shRNAs (shSerpinE2#15 or shSerpinE2#16). Stable cell populations were thereafter lysed and RNA isolated to determine *serpinE2 *or *β2MIC *gene expression by Q-PCR. The relative level of each mRNA was calculated using the standard curve method and normalized to the corresponding *β2MIC *mRNA level. **B- **LoVo or HCT116 cells expressing either shTGFP or shSerpinE2 (#16) were seeded in a 6-well plate at 200 000 cells per well. Cells were harvested, clumps disrupted using a syringe and counted. Values are means of 4 experiments ± SE. **C- **Cell populations were cultured in soft agarose for 2-3 weeks before MTT staining. The number of colonies was determined using Image J software. The number of shTGFP-expressing cells (control) was set at 100%. Results are the mean ± SE of at least 3 independent experiments. **D- **Phase-contrast microscopy of foci from two-week post-confluent shTGFP and shSerpinE2(#16)-expressing LoVo cells stained with crystal violet. *, significantly different from shTGFP cell populations (set at 1) at p < 0.05 (Student's *t *test); **, significantly different from control at p < 0.002 (Student *t *test).

We next investigated the effect of serpinE2 knockdown on anchorage independent growth and cell migration after downregulation of *serpinE2 *gene expression by RNA interference in HCT116 and LoVo cells. As shown in Figure 4A, *serpinE2 *mRNA were significantly reduced by respectively 37% and 88% in LoVo cells expressing shSerpinE2(#15) or shSerpinE2(#16) and by 77% and 92% in HCT116 expressing shSerpinE2(#15) or shSerpinE2(#16); conversely, expression of the control shRNA (shTGFP) had no effect on endogenous *serpinE2 *expression (data not shown). Again, the proliferation rate of these cell populations was assessed when cultured on plastic. No difference was observed in the proliferation rate of subconfluent cells when serpinE2 expression was downregulated (Figure 4B). We then verified whether the reduction in *serpinE2 *expression alters the ability of colon cancer cells to form colonies in soft agarose. As shown in Figure 4C, expression of both shRNA against SerpinE2 (#15 and #16) decreased the ability of HCT116 and LoVo cells to form colonies in soft agarose. Of note, shSerpinE2(#15) which was less efficient than the shRNA (#16) to reduce *serpinE2 *gene expression (Figure 4A) was also less efficient to reduce colony formation. This indicates that serpinE2 controls anchorage-independent growth of human colon carcinoma cells. Additionally, as observed in caMEK-expressing IECs, the size of foci formed at post-confluency was significantly decreased in serpinE2-depleted LoVo cells (Figure 4D).

**Figure 5:**
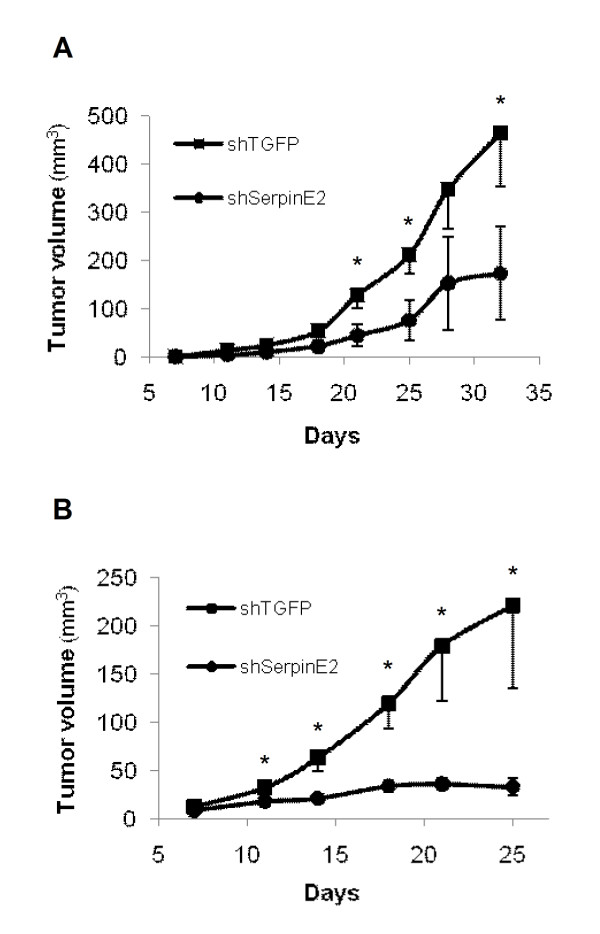
**Down-regulation of serpinE2 expression in human colorectal cancer cells inhibits tumor growth in nude mice**. **A and B- **The growth of tumors (mm^3^) over time was measured after s.c. injection of 1 × 10^6 ^cells (HCT116: left panel; LoVo: right panel). The results represent the mean tumor volume obtained from two independent experiments in which at least five mice were injected for each cell line. Tumor volumes were determined by external measurement (V = (d^2 ^× D)/2). Data are the means ± SE of five mice. *, significantly different from HCT116- or LoVo-shSerpinE2 (#16) cells at * p < 0.05.

The tumorigenicity of colorectal cell lines was next assessed after subcutaneous (s.c.) injection into the flank of nude mice. As shown in Figure 5A and 5B, HCT116 and LoVo cell lines induced palpable tumors with a short latency period of respectively 15 and 10 days after their injection. More importantly, downregulation of serpinE2 expression with shSerpinE2(#16) in these cell lines severely impaired their capacity to grow as tumors in nude mice.

**Figure 6:**
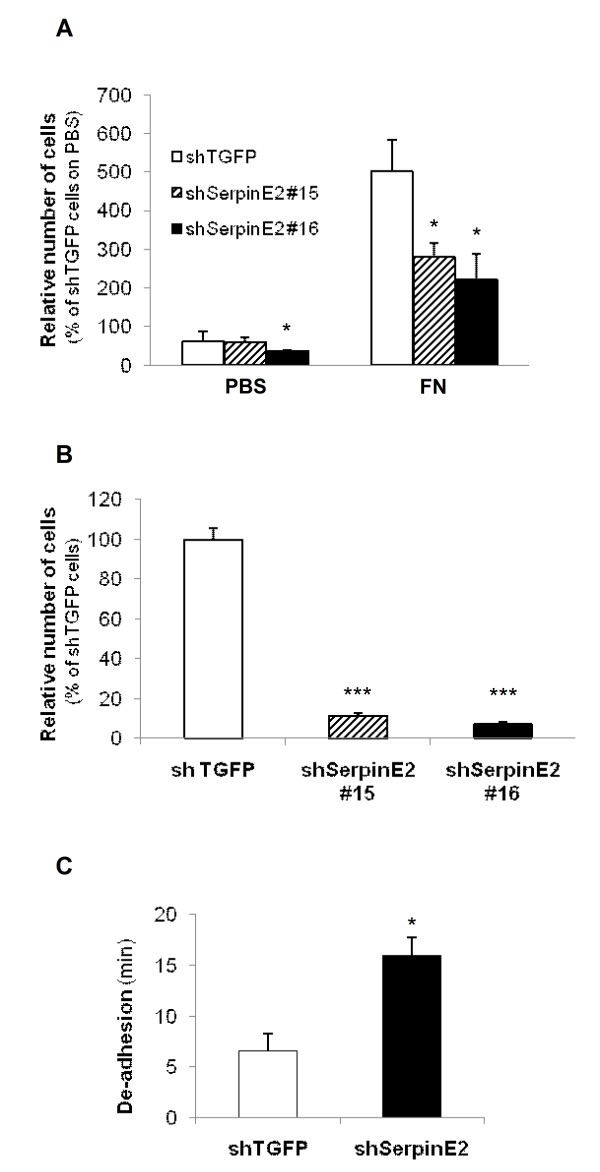
**SerpinE2 silencing modulates migration, invasion and adhesion of colorectal cancer cells**. **A- **Migration of shTGFP- and shSerpinE2(#15 or #16)-expressing LoVo cells to the undersurface of the polycarbonate membrane of Boyden chambers coated with fibronectin (FN) or vitronectin (data not shown) was evaluated 24 h after seeding, in presence of 20 mM hydroxyurea. The number of cells was counted in five fields and the experiments were performed in triplicate. The number of shTGFP cells that had migrated was set at 100%. **B- **Invasion of shTGFP- and shSerpinE2(#15 or #16)-expressing LoVo cells was studied using Matrigel-coated Transwells during 48 h. Thereafter, cells were fixed and stained with DAPI solution. The number of cells was counted in five fields and the experiments performed in duplicate. The number of shTGFP cells which had migrated was set at 100%. **C- **Time required for complete de-adhesion of shTGFP- and shSerpinE2(#16)-expressing LoVo cells after addition of trypsin while rocking at 100 rpm. *, significantly different from shTGFP at p < 0.05 (Student's *t *test). ***, significantly different from control at p < 0.0001 (Student *t *test).

Finally, *in vitro *transwell migration assays were performed to verify the importance of serpinE2 in colon carcinoma cell migration. As illustrated in Figure 6A, serpinE2 deficiency significantly reduced HCT116 (not shown) and LoVo cell migration to the undersurface of the membrane coated or not with fibronectin or vitronectin (data not shown). The net effect of serpinE2 knockdown was also determined on invasion by using BD Biocoat Matrigel invasion chambers, in presence of hydroxyurea. As shown in Figure 6B, the capacity of LoVo cells to invade Matrigel was also altered by serpinE2 silencing

To test the hypothesis that this altered migration and invasion capacity could result from a defect in cell adhesion, adhesion strength to the substrate was examined for control and shSerpinE2(#16)-expressing LoVo cells. Using a trypsin-mediated de-adhesion assay, downregulation of serpinE2 significantly delayed LoVo cell detachment after trypsinization (Figure 6C), suggesting that serpinE2 expression decreases adhesion of colorectal carcinoma cells to the substrate.

### *SerpinE2 *gene expression is up-regulated in human colorectal cancers

**Figure 7:**
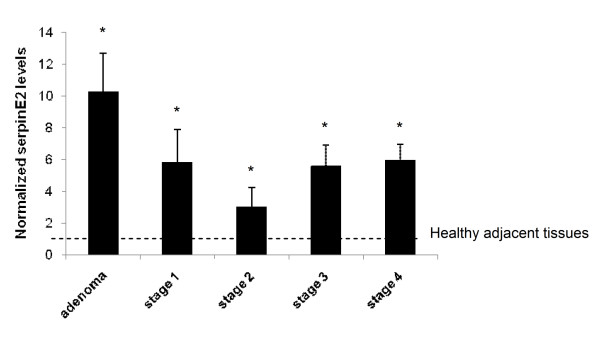
***SerpinE2 *gene expression is up-regulated in human colorectal tumors**. Relative *serpinE2 *mRNA levels were determined by Q-PCR of human advanced adenomas and cancers compared to the adjacent healthy tissue (set at 1). mRNA levels were normalized to *β2MIC *mRNA levels. Data presented in are means ± SE of 5 to 19 biopsies per stage. *, significantly different from adjacent healthy tissue at p < 0.05 (Student's *t *test).

We next analyzed *serpinE2 *gene expression in a series of human paired specimens (resection margins and primary tumors) by Q-PCR analysis. As shown in Figure 7, mRNA levels of serpinE2 were markedly increased in human adenomas in comparison to healthy adjacent tissues. Furthermore, serpinE2 expression was also significantly enhanced in colorectal tumors, regardless of tumor stage and grade.

## Discussion

We and others have recently reported that expression of a constitutively active mutant of MEK1 in normal intestinal epithelial cells is sufficient to induce growth factor relaxation for DNA synthesis, morphological transformation, growth in soft agar, epithelial to mesenchymal transition and to promote tumor invasion and metastasis [3,4,14,15]. Thus, these data argue that a key role of sustained MEK activity resulting from the constitutive activation of KRAS or BRAF in colorectal carcinoma cells may be to provide signals inducing not only proliferation, but also transformation and tumorigenesis. However, in spite of the obvious role of MEK/ERK kinases in the induction and regulation of intestinal epithelial cell tumorigenesis, little is known as to the molecular mechanisms by which this signaling achieves such functions. In the present study, we show that *serpinE2 *gene is a MEK1 target in intestinal epithelial cells and that serpinE2 expression and secretion correlate with both MEK1 activity and intestinal epithelial cell transformation. Moreover, targeting of *serpinE2 *by mRNAi in human colorectal cancer cell lines decreased anchorage independent growth, migration, invasion as well as tumor formation in nude mice. Accordingly, we found an upregulation of *serpinE2 *mRNA levels in human adenomas and colorectal cancer tissues as compared to corresponding normal tissues.

Oncogenic mutations in *KRAS *or *BRAF *occur frequently in colorectal cancer and aberrant signaling through the ERK pathway has been correlated with both initiation [38] and progression [13] of CRC. Interestingly, *KRAS *and *BRAF *mutations seem to be mutually exclusive [39,40], suggesting that they may have similar functions. These oncogenes primarily signal through the MEK/ERK pathway [41,42]. Upon phosphorylation by MEK1/2, ERK1/2 translocate to the nucleus and phosphorylate various transcription factors regulating gene expression [43]. Therefore, in order to define the genetic changes induced by persistent MEK activation, we and others [4,15] have utilized oligonucleotide microarrays to determine which genes are regulated following the constitutive activation of MEK in normal intestinal epithelial cells. Our results revealed that *serpinE2 *gene was the gene mostly induced by activated MEK in intestinal epithelial cells. This observed altered level of expression of *serpinE2 *transcript was also noted in microarray analyses performed by Voisin and colleagues [15]. In the present study, we were able to confirm that *RAS-*, *BRAF- *and *caMEK*-transformed intestinal epithelial cells express and secrete serpinE2. Furthermore, serpinE2 expression was rapidly enhanced (in 1 h) upon induction of oncogenic BRAF in normal intestinal epithelial cells, suggesting an early involvement of this protein in cell transformation. Of note, expression of serpinE2 in human colorectal cancer cell lines was shown to be dependent, at least in part, of endogenous activities of MEK/ERK. Other oncogenic pathways have been previously associated with induction of serpinE2 expression. Indeed, the very oncogenic receptor tyrosine kinase MET was also shown to promote *serpinE2 *gene expression in a xenograft colon tumor model [44]. Additionally, PTEN deletion has been reported to up-regulate *serpinE2 *expression in MEF cells [45] and serpinE2 was shown to be overexpressed in cells transformed by adenovirus type 12 [46]. Taken together, these results indicate that *serpinE2 *gene expression could be induced by different oncogenic pathways, emphasizing that this protein may be important in tumorigenesis.

Our results also led to the demonstration that serpinE2 contributes to transformation induced by activated MEK1 and to human colorectal carcinoma cell growth and migration. In agreement with the present study, data on serpinE2 expression in human cancer indicate that serpinE2 levels are elevated in pancreatic tumors [27], breast tumors [28], liposarcomas [31] and oral squamous carcinomas [30]. Accordingly, we found a significantly higher level of *serpinE2 *mRNA when comparing affected tissues from advanced adenomas and carcinomas to adjacent healthy tissues. These results are in agreement with the study of Selzer-Plon et al. who recently reported that *serpinE2 *mRNA levels increase both at the transition between normal tissue and adenomas with mild/moderate dysplasia and again at the transition between severe dysplasia and colorectal cancer [32]. In addition, no significant difference was observed when comparing s*erpinE2 *mRNA levels in primary cancers classified into different TNM stages. Taken together, the above results suggest that enhanced serpinE2 expression may be implicated in tumor progression in colorectal tissue.

Although there is some evidence in the literature suggesting that serpinE2 may play a role in carcinogenesis, the precise function of this serpin in cancer still remains elusive. Through its ability to reduce proteolysis, this serine protease inhibitor is predicted to impair extracellular matrix degradation and consequently cancer cell invasion and metastasis. However, overexpression of serpinE2 appears to enhance the invasive potential of pancreatic tumors in xenograft models [27]. Recently, using mammary tumor models, it has been reported that serpinE2 stimulates metastatic spread of mammary tumors [47]. In addition, an analysis of 126 breast cancer patients revealed that patients with breast tumors showing elevated serpinE2 levels also had a significantly higher probability of developing lung metastasis [47]. Finally, serpinE2 has recently been shown to promote lymph node metastasis in a testicular cancer model [48]. Thus, increased function of serpinE2 appears to be associated with enhanced migration and metastasis. However, the biological roles of serpinE2 in colorectal carcinoma have never been studied. Herein, the present results show that endogenous expression of serpinE2 in rodent transformed intestinal epithelial cells and human CRC cells is correlated with enhanced cell migration and invasion abilities. The molecular mechanism by which serpinE2 modulates motility remains unknown. It is possible that serpinE2 may enhance signaling cascades mediating motility. In this regard, serpinE2 has recently been reported to stimulate ERK signaling by binding LRP-1 [47] or syndecan-1 [49]. However, preliminary results (data not shown) indicate that the phosphorylated levels of Akt and ERK1/2 were not affected following serpinE2 depletion in colon carcinoma cells. Alternatively, shSerpinE2-expressing cells may have a reduced migratory capacity which could result from a defect in cell adhesion. Indeed, typical cell movement across a two-dimensional substrate can be divided into three concerted steps: membrane protrusion, cell traction, deadhesion and tail retraction. Adhesion at the leading edge and deadhesion at the rear portion of cells are required for protrusion and tail retraction, respectively [50]. As cellular migration and cellular adhesion are intimately related, changes in one could be expected to result in changes in the other. Binding of type-1 plasminogen activator inhibitor (PAI-1), the phylogenetically closest relative of serpinE2, to cell surface uPA promotes inactivation and internalization of adhesion receptors (e.g. urokinase receptor and integrins) and leads to cell detachment from a variety of extracellular matrixes [51]. Recently, serpinE2 has been shown to also induce cell detachment from a variety of extracellular matrix proteins such as vitronectin, fibronectin and type-1 collagen in an uPA/uPAR-dependent manner [52]. Interestingly, serpinE2 has been reported to co-localize with fibronectin [34] and to interact with vitronectin [35]. Accordingly, we observed herein that the downregulation of serpinE2 significantly delayed colorectal carcinoma cell detachment after trypsinization, suggesting that serpinE2 expression does decrease adhesion and promote detachment of colorectal carcinoma cells. Moreover, we have recently demonstrated that uPA expression levels are enhanced in MEK1-transformed intestinal epithelial cells [14]. Further experiments are hence necessary to clearly identify the molecular mechanisms involved in the deadhesive effects of serpinE2.

## Conclusion

Our study identifies the serine protease inhibitor serpinE2 as a novel target of ERK signaling involved in human colorectal tumorigenesis. The strong expression of serpinE2 in human adenomas suggests that this secreted protein might be a potential blood biomarker for early diagnosis of tumors in the colon and the rectum. While further studies are needed to pinpoint the molecular mechanisms by which serpinE2 regulates tumor cell growth and migration, the present study provides novel fundamental insights into the function of serpinE2 in colorectal cancer progression. Hence, serpinE2 may also be a potential therapeutic target for cancer treatment.

## Methods

### Materials

The anti-bovine serpinE2 antibody was previously characterized [53]. The antibody recognizing β-actin was obtained from Chemicon International (Billerica, MA). Antibodies recognizing phospho-ERK1/2 (Thr202/Tyr204) #9101 and total ERK were from Cell Signaling Technology (Danvers, MA). The MEK inhibitor U0126 was from Calbiochem-Novabiochem Corp. (San Diego, CA). Human plasma-derived fibronectin and vitronectin were from R&D systems (Minneapolis, MN). MTT was purchased from Invitrogen (Carlsbad, CA). Other materials were obtained from Sigma-Aldrich unless stated otherwise.

### Cell culture

The rat intestinal epithelial crypt cell line IEC-6 stably overexpressing pLXIN-wtMEK or -caMEK were previously characterized [3] and cultured as described [14]. These cell populations were generated after viral infection of wtMEK and caMEK cloned in the retroviral vector pLXIN. The caMEK-expressing cells formed foci at post-confluency, in contrast to pLXIN- and wtMEK-expressing epithelioid cells which formed a monolayer of contact-inhibited cells. Foci from post-confluent caMEK-expressing cells were therefore retrieved by aspiration with a pipette and pooled as one caMEK-expressing cell population. The majority of experiments described herein was performed with this caMEK-expressing cell population and compared to pLXIN and wtMEK-expressing cell populations unless otherwise stated. This strategy was repeated independently three times with other IEC-6 cell cultures and similar results were obtained with all caMEK-expressing cell populations. The IEC6 wtMEK and caMEK were cultured in DMEM containing 5% FCS. The IEC-6-BRAF:ER population was obtained by retroviral infection of IEC-6 cells as previously described [14] with the plasmid encoding the fusion protein consisting of full-length human BRAFV600E linked to the T1 form of the human estrogen receptor hormone-binding domain and selection of cells resistant to blasticidin S (5 mg/ml). The population displayed strong stimulation of ERK1/2 activity upon β-estradiol or tamoxifen addition as previously reported [54]. IEC6 BRAF^V600E ^cells were cultured in DMEM without phenol red, supplemented with 5% charcoal stripped FCS (Valley Biomedical, Winchester, VA, USA). The transformed cell line *Ha-ras*IEC-6, previously characterized [55], was cultured in DMEM containing 5% FCS. The cell line Caco-2/15 was obtained from Dr A. Quaroni (Cornell University, Ithaca, NY) and cultured in DMEM containing 10% FCS, as described previously [56]. The colon carcinoma cell lines HCT116 and HT29 were obtained from ATCC (CCL-247 and HTB-38) and cultured in McCoy's medium (Wisent) containing 10% FCS. The colon adenocarcinoma cell lines Lovo (CCL 229, ATCC) and SW480 (CCL 228, ATCC) were respectively cultured in Ham's F12 medium containing 10% FCS and in DMEM containing 10% FBS. The colon adenocarcinoma cell lines DLD-1 (CCL-21) (kindly obtained from Dr F. Boudreau, Université de Sherbrooke, QC, Canada) and Colo205 (CCL-222, ATCC) were cultured in RPMI medium containing 10% FCS. The colorectal carcinoma cell line T84 (CCL-248) was cultured in DMEM-Ham's F-12 (50:50) containing 10% FBS.

### Microarray analysis

Total RNAs were extracted from newly confluent IEC-6 cells stably expressing wtMEK or caMEK with the RNeasy kit (Qiagen inc., Mississauga, ON, Canada). For microarray analysis, 10 μg of RNA were used for cDNA synthesis, followed by *in vitro *transcription to generate biotin-labeled cDNAs with a T7 promoter primer having a poly(T) tail for subsequent hybridization. The resulting product was hybridized and processed with the Rat Genome RAE230 2.0 Array GeneChip system (Affymetrix). Three independent experiments were carried out for each condition. Data analysis, normalization, average difference and expression for each feature on the chip were performed using Affymetrix Microarray Suite 5.0 (MAS5) with default parameters (Microarray platform, McGill University and Genome Quebec Innovation Centre). Gene classification according to cellular processes was performed with the Database for Annotation, Visualization and Integrated Discovery (DAVID) http://apps1.niaid.nih.gov/david.

### Animals

CD1 nu/nu mice were purchased from Charles River Laboratory (Montréal, Canada). All experiments were approved by the animal research committee of the Faculty of Medicine and Health Sciences of the Université de Sherbrooke.

### Human biopsies

Samples of colon tumors and paired normal colon tissues (at least 10 cm from the tumor) were obtained from patients undergoing surgical resection. Patients did not receive neoadjuvant therapy. Tissues were obtained after patient's written informed consent, according to the protocol approved by the Institutional Human Subject Review Board of the Centre Hospitalier Universitaire de Sherbrooke. Paired tissues were frozen in liquid nitrogen within 15 minutes from resection as recommended by the Canadian Tumor Repository Network http://www.ctrnet.ca and stored in liquid nitrogen until total RNA extraction. Clinical and pathological informations were obtained from medical records. Adenoma samples were endoscopically unresectable and defined as advanced because of their size larger than 1 cm or by the presence of high-grade dysplasia or villous component. Patient's cancers were histologically classified and graded according to overall TNM staging criteria (based on Tumor-, lymph Node- and Metastatic- status).

### Reverse transcription PCR

Total RNA was extracted from cultured cell lines or human colorectal adenoma or tumors and their respective adjacent healthy mucosa using the RNeasy mini kit (Qiagen, Canada) using gDNA Eliminator spin columns or an on-column DNAse I digestion step (human samples). Reverse transcription and PCR were performed using AMV-RT (Roche Diagnostics, Canada) and Taq DNA polymerase (Qiagen, Canada) according to the manufacturer's instructions. Real-time PCR analysis (QPCR) was performed using a Light-Cycler apparatus (Roche Diagnostic, Canada) as previously described [14]. Target expression was quantified relatively to β-actin or TBP (rat) or β-2-microglobulin (β2MIC) (human) [57] expression. Primers rSerpinE2 forward: CCCTACCATGGTGTGAGAGCAT; rSerpinE2 backward: GCCTTTGACGGTTCAAACAT; Primers: mSerpinE2 forward: CCCTACCATGGTGAGAGCAT; mSerpinE2 backward: GCCTTTGATGGCTCAAACAT; hSerpinE2 forward: AATGAAACCAGGGATATGATTGAC; hSerpinE2 backward: TTGCAAGATATGAGAAACATGGAG; β-actin forward: ACCACAGCTGACAGGAAATCG; β-actin backward: AGAGGTCTTTACGCATGTCAACG; TBP forward: TTCAGTTCTGGGAAAATGGTG; TBP backward: GGAGAACAATTCTGGGTTTGA; GAPDH forward: ATGGCCTTCCGTGTTCCTAC; β2MIC backward: TCGCGCTACTCTCTGTTTCTG.

### Immunoblotting

SDS-PAGE and immunoblot analyses were performed as previously described [3]. For culture medium analysis, subconfluent cells cultured in a 100 mm dish were incubated overnight with a fresh 8 mL of serum free medium after which the medium collected and cells harvested in a lysis buffer containing 150 mM NaCl, 1 mM EDTA, 40 mM Tris-HCl, pH 7.6, 1% NP-40 supplemented with protease inhibitors (0.1 mM PMSF, 10 μg/ml leupeptin, 1 μg/ml pepstatin, 10 μg/ml aprotinin). A volume of medium proportional to the total amount of protein in the cell lysate was passed through an Amicon(r) Ultra-4 centrifugal filter unit (10,000 NMWL; Millipore). Laemmli buffer was added to the retentate and boiled for 5 min. Protein concentrations were determined using the bicinchoninic acid (*BCA*) assay (Pierce) with bovine serum albumin as standard.

### RNA interference

*Rat IEC6 cells*: shRNA oligonucleotides were designed according to Ambion guidelines (technical bulletin no. 506), annealed and cloned into pLenti6-U6 expression vector [14] between BamHI and XhoI sites. siRNA sequences were AGGAACCATGAATTGGCAT (SerpinE2) or GGCAGTTCAGACAGATTAA (Scrambled) and TTCAAGAGA as loop sequence. *Human LoVo and HCT116 cells*: To downregulate *serpinE2 *expression, mission(r) shRNA Bacterial Glycerol Stock were purchased from Sigma-Aldrich (NM_006216). Sequences used were CCGGCCTCGTCAACGCAGTGTATTTCTCGAGAAATACACTGCGTTGACGAGGTTTTTG (shSerpinE2#15), CCGGGAACACAAAGAAACGCACTTTCTCGAGAAAGTGCGTTTCTTTGTGTTCTTTTTG (shSerpinE2#16) and CCGGCGTGATCTTCACCGACAAGATCTCGAGATCTTGTCGGTGAAGATCACGTTTTT (control: TurboGFP(tm) (shTGFP)). All lentiviruses were produced and used for cell infection according to Invitrogen recommendations (ViraPower Lentiviral Expression System, instructions manual). In each experiment involving lentiviruses, *OAS1 *gene expression was analyzed by Q-PCR analysis. *OAS1 *(2'5'-oligoadenylate synthetase) is a classic interferon target gene and has been recommended as a key test for interferon induction before attributing a particular response to the gene that is targeted [58]. No induction of *OAS1 *expression was detected in the experiments involving lentiviruses infection (data not shown).

### Cell proliferation assays

All experiments were performed starting with cell populations after at least 14 days post-selection and subsequently plated for growth assay in 6-well plates at a concentration of 100 000 cells/well for IEC-6 and 200 000 cells/well for HCT116 and LoVo. Cell growth was measured during 7-8 days using a Cell particle counter.

### Focus formation assays

Parental IEC-6 cells were seeded into 30-mm dishes in triplicate. Cells were grown to confluence and confluent monolayers were adapted over a week-long period to DMEM/5%FBS before seeding of caMEK-expressing cells at high density (5000cells/well). These cells were then grown by forming foci and maintained in culture for 14-20 days. Thereafter, cells were washed twice with 1× PBS and fixed with methanol for 1 min. Methanol was removed and 1% crystal violet solution was added for 2 min. Excess dye was carefully removed with water and plates were dried at room temperature. Analysis was performed by counting the number and size of the foci using Image J software. Resulting data were analyzed by Student's *t *test.

### Soft agarose

Concentrated DMEM-2X without phenol red was prepared from powder (Wisent, Qc Canada) according to manufacturer's instructions, except for using half of the recommended volume of water. The medium was sterilized by 0.22 μm filtration and complemented with 10% (IEC6) or 20% (LoVo and HCT116) FBS. Pre-warmed DMEM-2X was mixed 1:1 with autoclaved 1.4% agarose type VII kept at ≈42°C and 6-well dishes were pre-coated with 1 ml/well. Cells were added to the DMEM-agarose mix at 10000 cells/mL (IEC6) or 5000 cells/mL (LoVo and HCT116) and seeded at 2 mL/well. Plates were allowed to solidify under the hood and then placed at 37°C and 5% CO_2_. Fresh DMEM without phenol red supplemented with 5% (IEC6) - 10% (LoVo and HCT116) FBS was added on the surface of the agarose every 2-3 days. After 2-3 weeks, colonies were stained by adding 500 μL of PBS containing 0.5 mg/mL MTT on the surface of the agarose and incubated 2 hours at 37°C and 5% CO_2_. Images were acquired using an AlphaImager camera (Alpha Innotech Corporation) and colonies counted using ImageJ software.

### Migration and invasion assays

Cell migration was assessed using Transwell(r)-24 well permeable support (8.0-μm pored polycarbonate membranes (Corning)). The bottom face of membranes was coated or not with 10 μg/μL fibronectin or vitronectin for 1 hour at 37°C and then rinsed with PBS. Thereafter, 3000cells in 200 μL of serum-free medium were seeded into the upper chamber and culture medium containing 5% FBS was placed into the lower chamber as chemoattractant agent. Cells were allowed to migrate for the next 24 h (IEC-6) or 48 h (LoVo) in the presence of 2 mM hydroxyurea in both chambers to prevent cell proliferation. Non-migrating cells were removed with 2 cotton swabs, while migrating cells were fixed for 2 min with methanol and stained with DAPI for manual counting under the microscope. Invasion assays were conducted using BD Matrigel(tm) Invasion Chamber 24-well plate 8.0 micron according to the manufacturer's instructions. Briefly, plates were thawed at room temperature for 30 min and then Matrigel humidified with HAM'S F12 culture medium for at least 1 hour at 37°C and 5% CO_2_. Thereafter, 6000cells in 200 μL of serum-free medium were seeded into the upper chamber and culture medium containing 5% FBS was placed into the lower chamber as chemoattractant agent. Cells were allowed to migrate for the next 48 h in the presence of 2 mM hydroxyurea in both chambers to prevent cell proliferation [59]. Cells were then processed as described above for migration assays.

### Xenografts into nude mice

A total of 1 × 10^6 ^cells suspended in 0.1 ml DMEM were injected into the dorsal subcutaneous (s.c.) tissue of 5-week-old female nude mice CD1 *nu/nu *(Charles River, Canada). Both control and experimental cell lines were contralaterally injected into each individual animal. Tumor volume was determined by external measurement according to published methods (d^2 ^x D)/2 [60].

### De-adhesion assays

Subconfluent cells were rinsed twice with PBS before addition of 500 μL of 0.25% trypsin/0.1 mM EDTA (Wisent, Qc, Canada) per well of a 6-well dish. Plates were rocked at 100 RPM at room temperature until cells were completely detached.

### Data Presentation and Statistical Analysis

Densitometric analyses were performed using Image J software and were carried out in RT-PCR analyses shown in Figure 1. Results shown in the graphs were analyzed by the Student's *t *test. Differences were considered significantly different at *p *< 0.05, unless otherwise stated. Results shown are the mean of at least three independent experiments.

## Competing interests

The authors declare that they have no competing interests.

## Authors' contributions

MJB performed the microarray analysis. SB performed the molecular genetic studies, generated shRNA against serpinE2. SB and EL characterized the phenotype of all shSerpinE2-expressing cell populations (Figure 2, 4 and 6). SB also analyzed *serpinE2 *mRNA levels in human colorectal tumors and cell lines (Figure 3A and 6). Finally, SB drafted the manuscript. VD performed the first RT-PCR and Western blot analyses demonstrating the induction of serpinE2 by oncogenic MEK1 and Ras (Figure 1A, B, D and 1E). SC generated cells expressing the inducible BRAF:ER fusion protein stimulated with 4-hydroxytamoxifen (4-OHT) (Figure 1C). EL participated in the design of the study and performed Q-PCR analyses (Figure 3B and 4A). NR conceived the study, and participated in its design and coordination and helped draft the manuscript. All authors read and approved the final manuscript.
